# Factors influencing late antenatal booking in Tshwane District: Pregnant women’s perceptions

**DOI:** 10.4102/phcfm.v17i1.4870

**Published:** 2025-05-29

**Authors:** Kagiso P. Tukisi, Vuyo D. Dlakude, Sakhile I. Hlatshwayo, Fezeka Dlamini

**Affiliations:** 1 Department of Nursing, School of Healthcare Science, Sefako Makgatho Health Sciences University, Pretoria, South Africa

**Keywords:** antenatal care, antenatal booking, pregnancy, pregnancy outcome, maternal and neonatal outcomes

## Abstract

**Background:**

Antenatal care (ANC) is a branch of primary health care service universally accessible for promoting positive maternal and neonatal outcomes globally. Pregnant women are encouraged to initiate ANC as soon as pregnancy is diagnosed. Early ANC allows a series of diagnostic procedures and investigations to exclude early, potential and actual pregnancy risks. However, the rate of late initiation of ANC remains high.

**Aim:**

To explore and describe factors influencing late antenatal booking based on pregnant women’s perceptions in selected antenatal clinics in the Tshwane district.

**Setting:**

The study took place at the two selected facilities rendering ANC to the public in Tshwane district.

**Methods:**

A qualitative, explorative, descriptive and contextual research design was followed, and 10 purposively sampled pregnant women attended semi-structured interviews. Collaizi’s descriptive method was used to analyse and organise data into themes and categories.

**Results:**

Although the participants had some awareness of ANC and the benefits attached, there were hindrances to the early seeking of ANC. The participants brought to light the factors that hinder early seeking of ANC under three themes: Theme 1 listed the patient-related factors; Theme 2 detailed ANC routine factors; and lastly, Theme 3 described midwives-related factors.

**Conclusion:**

The information dissemination methods of reproductive health and childbirth need to be revisited to ensure awareness and increase uptake of the ANC services.

**Contribution:**

The study findings have the potential to guide policymakers in addressing the factors that hinder the uptake of ANC as perceived by pregnant women – the primary consumers of the service. Additionally, the uptake of ANC may contribute to a decline in maternal and neonatal mortalities.

## Introduction

Antenatal care (ANC) is a cornerstone of maternal health that significantly improves maternal and neonatal outcomes globally. Antenatal care service is a recommendation from the World Health Organization (WHO) for all pregnant women to be attended by a midwife throughout pregnancy, in collaboration with an obstetrician in case of high-risk pregnancies.^1^ The need for monitoring of pregnancy stems from the physiology of pregnancy, which may result in minor disorders and serious complications, which in turn may result in deleterious maternal and neonatal outcomes.^2^ Thus, the WHO has introduced Basic Antenatal Care (BANC) and Basic Antenatal Care Plus (BANC Plus) as a programme for midwives to assess, diagnose and monitor pregnant women in primary health care facilities, including midwives-led obstetric units (MOU).^3^

The BANC and BANC Plus aim to ensure universal access to safe and efficient ANC service, including in resource countries, resource-deficient.^3^ Basic Antenatal Care and BANC PLUS comprise initial and subsequent visits.^3^ An ANC booking refers to a pregnant woman’s first and initial visit to the midwife or obstetrician to seek ANC service.^4^ Basic Antenatal Care has four visits typically designed for low-risk pregnancies, and they are scheduled as follows: first visit – before 12 weeks; second visit – at 20 weeks; third visit – at 26–28 weeks; and fourth visit – at 34–36 weeks respectively.^5^ The subsequent visit in BANC plus refers to the follow-up visits scheduled at 20, 26, 30, 34, 36, 38 and 40 weeks of gestation, respectively.^4,5^

According to the BANC guidelines, ANC should be initiated as soon as the woman experiences their first amenorrhea and before 12 completed weeks of gestation for the visit to be classified as an early initial visit.^3^ An early initial visit is recommended to allow the pregnant woman to receive folic acid supplementation to aid with the replication of cells during organogenesis and prevent foetal neural tube defects.^6^ Additionally, early initiation of ANC allows the midwives to relook the treatment of existing medical conditions, which could have teratogenic effects or have the potential to be exacerbated by the physiology of pregnancy.^7^ Furthermore, the literature suggests that early initiation of ANC allows the pregnant woman to be counselled on lifestyle, which may lead to the cessation of smoking, thereby reducing the risk of intrauterine growth restrictions (IUGR), and of alcohol consumption, which may help prevent foetal alcohol syndrome (FAS).^8,9^

Although the importance of early initiation of ANC is amplified in the literature, the rate of late initiation of ANC is 58.6% globally, suggesting that about 43% of women initiate ANC early.^10,11^ This suggests that late initiation of ANC remains prevalent and generally high. According to the literature, the rate of early initiation of ANC is 38% in sub-Saharan countries.^12^ In South Africa, 68.3% of women initiated their ANC before 20 weeks of gestation, a marked decline from 69.7% recorded in 2019.^13^

According to the literature, late ANC delays the early detection of some of the potential and actual problems, inadvertently delaying prompt management and leading to adverse outcomes.^14^ Anaemia in pregnancy is reported as a common condition that requires close monitoring and management, as it is exacerbated by haemodilution, which is part of the physiological changes within the cardiovascular system.^15^ Consequently, anaemia in pregnancy poses a risk of foetal compromise and placenta abruption with complicated postpartum haemorrhage.^15^ Another serious condition that requires women to initiate ANC early is pregnancy-related hypertensive disorders to ensure pregnant women receive prophylactic calcium gluconate.^16^

The complications resulting from delayed ANC are avoidable adverse outcomes that could have been managed if diagnosed promptly.^17^ Therefore, delay in seeking ANC is classified as an inappropriate response to pregnancy by the perinatal problem identification programme (PPIP).^17^ Studies revealed that 72.9% of the 924 maternal death cases they analysed were associated with delay in seeking ANC.^18^ Delayed ANC can disenable the attainment of the Sustainable Developmental Goal (SDG) 3.1, aiming to reduce maternal mortalities to less than 70 per 100 000 births.^19^ Additionally, delayed ANC may affect the set target of reducing the neonatal mortality rate to less than 12 per 1000 live births by 2030, according to SDG 3.2.2.^19^

To improve the rate of early ANC booking, the barriers and challenges surrounding the ANC as perceived and experienced by pregnant women should be addressed. Failure to address these factors will lead to continued late ANC booking with its associated adverse and avoidable maternal and neonatal outcomes. The study explores and describes the factors influencing late ANC booking as perceived by pregnant women. Understanding the factors that hinder the initiation of such an important care from the consumers of the service will provide insights that will guide the rollout plans for ANC. Addressing the existing factors will improve access to the ANC, and the aims of the ANC, which include improving maternal and neonatal outcomes, will be achieved.

## Research methods and design

### Study design

A qualitative, explorative and descriptive research design was followed to gain an understanding of the factors influencing late ANC booking through the lens of pregnant women in the Tshwane district.^20^

### Study settings

The research was conducted in three selected ANC facilities in the Tshwane district, in the Gauteng Province, South Africa. The selected facilities comprise two Community Health Centres (CHC) that render 8 h ANC from 07:00 to 16:00 on weekdays. The third facility is an MOU, which provides 24 h health services. In all three facilities, the ANC service is primarily the midwives’ responsibility to the communities residing in the northern zone of Region 1 in the City of Tshwane.

### Population

The study targets pregnant women attending antenatal clinics in the Tshwane district. This accessible population represents the group of women who are realistically within the researchers’ reach and can actively participate in the study. By selecting pregnant women from these local clinics, the researchers gathered direct, first-hand information about their experiences, perceptions and challenges related to ANC.

The research design acknowledged the difference between the ideal target population (all pregnant women) and the accessible population (pregnant women at selected Tshwane district clinics).^21^ This approach allowed the researchers to collect meaningful data while recognising the practical limitations of conducting comprehensive research. The ultimate objective remains to generate insights that can potentially be applied more broadly to improve understanding and awareness of ANC services for pregnant women.

### Sampling strategy

A non-probability, purposive sampling method was utilised to select a sample of 10 pregnant women who initiated their ANC late from a population of pregnant women in the ANC facilities.^21^

### Inclusion criteria

To participate in the study, the participants had to meet the following inclusion criteria: Pregnant women who booked for the ANC after 12 weeks of gestation at any of the three selected facilities. The pregnant woman had to be 18 years of age to grant voluntary consent in line with section 71 of the *National Health Act 61* of 2003.^22^

### Data collection

Data collection refers to the systematic process of gathering and measuring information from various sources to answer research questions, test hypotheses or evaluate outcomes.^21^ During data collection, the researcher finds, selects and records specific information needed for a research study or project.^21^

Data collection took place between October and December 2024. The participants were invited to the study through the operational managers who were gatekeepers. The research information letter was presented to operational managers, who subsequently granted access to the participants. Health education sessions in the morning allowed the researchers to present the research scope and invite the prospective participants who met the inclusion criteria to Participate the study.

The researchers targeted the health education sessions to communicate research information and to avoid the interruptions of the ANC and inconveniencing the patients. Ten semi-structured interviews were conducted with pregnant women who met the inclusion criteria.^21^ Verbal and written consent for participation and recording of the interviews were obtained. The first three interviews were regarded as pilot interviews to test the simplicity of the research question and its ability to yield information pertaining to the study.^23^ The research question was unambiguous; consequently, there was no need to revise the question.^23^ The responses of the participants of the first three pilot interviews were consistent with the responses of other participants and were included in the main sample of 10 participants.

The interviews were conducted in the boardroom within the facilities away from other pregnant women and staff members which ensured privacy and freedom of expression. Interviews lasted between 40 and 60 min. An open-ended central question was asked for all the participants to allow for spontaneity of responses, and where necessary, the researcher used probing questions to gain in-depth information.^24^ The following was the central question which was followed by the probes:

- *What are the factors influencing the late antenatal booking among pregnant women in selected antenatal clinics in the Tshwane district?*

There were no language barriers encountered during data collection as all participants were literate and were able to express themselves and comprehend English. Data saturation was reached during the 7th participant and the researchers conducted three more interviews to confirm the redundancy of information.^24^ At the end of the interview, the researchers provided the ANC services such as health education and routine midwifery care ensuring that the participants do not miss the essential services for the day.

### Data analysis

We employed seven steps of Colaizzi’s Descriptive Phenomenological Method of data analysis to describe the factors influencing late ANC booking among pregnant women in Tshwane.^25^ Data were analysed as follows.

#### Step 1: Read the transcriptions to acquire a feeling for them

Following verbatim transcription, the researchers read the transcriptions several times to gain an understanding of the pregnant women’s perceptions of the factors influencing late antenatal booking. The researchers intentionally listened to the recordings and read the transcriptions intensively to immerse themselves in the data and extract significant statements about the ANC booking.

#### Step 2: Extract significant statements

The researchers reviewed each interview and extracted the statements which relate directly to the pregnant women’s perceptions of the factors influencing late antenatal booking.

#### Step 3: Formulate the meanings of each significant statement

The researchers referred to the transcriptions to formulate meanings from the hidden meanings underlying the significant statements from the pregnant women’s perceptions. The significant statements were clustered into categories and themes detailing the factors influencing late ANC booking among pregnant women in Tshwane.^25^

#### Step 4: Organise the formulated meanings into clusters of themes

The formulated meanings were then organised into themes detailing the factors influencing late antenatal booking.

#### Step 5: Integrate the results into exhaustive meanings

The results of the study on pregnant women’s perceptions of the factors influencing late antenatal booking were integrated to form theme clusters and themes to formulate exhaustive meanings for a full description of the factors influencing late antenatal booking.

#### Step 6: Formulate an exhaustive description

The theme clusters and themes that emerged from the formulated meanings were integrated to form an exhaustive description of the pregnant women’s perceptions of the factors influencing late antenatal booking.

#### Step 7: Validate

The research findings were discussed between the participants and the research supervisor to validate the themes for relevance to the research question. In addition, the independent coder was employed to identify the themes. The researchers and the independent coder held a meeting, and the findings were discussed and collated, and consensus was reached on the final themes.

### Trustworthiness

To ensure trustworthiness, we employed five criteria of Lincoln and Guba’s framework.^26^ Prior to the interviews, the researchers engaged the prospective participants during health education sessions in the mornings which helped to establish rapport and trust increasing the credibility of the study. At the end of the interview, the researchers provided a summary of the interview for each participant to validate their responses which served as member checking. The researchers coded all the interviews which ensured referential adequacy. A code-recorded data were used to ensure the consistency of analysed data over time which strengthened the dependability of the study.^26^ In addition, an independent coder was employed for the analysis of data and confirmation of themes. The transferability of the study was ensured by detailing a clear plan of research methods to follow in addressing the research problem. Researchers kept an audit trail of recordings of the interviews and verbatim transcriptions and used direct quotations from the participants.^26^ We provided a detailed description of the participants’ demographic data which increased the generalisability of the study.

### Ethical considerations

We were cognisant that the participants in our study were human subjects and adhered to the ethical considerations of health science research.^27^ Ethical clearance to conduct this study was obtained from the Sefako Makgatho Health Sciences University Research Ethics Committee (SMUREC) (No. SMUREC/H/266/2024:UG). The participants are pregnant women and were accessed through the gatekeepers to avoid coercion. The participants received detailed information pertaining to the study presented during health education sessions while waiting for the ANC service, which enabled the participants to make informed choices about participating.^27^ The inclusion and exclusion criteria were adhered to which ensured fairness in the selection of participants. We conducted the interviews in a closed boardroom, thus maintaining the privacy of participants. In addition, we generated codes specifically for discussion and publication of data to ensure participants’ anonymity.^27^ We kept the research documents and recordings in password-encrypted files accessible only to the researchers, further ensuring privacy and confidentiality.

## Results

The study included 10 participants (P1–P10) with the following demographic characteristics: The participants ranged in age from 18 to 38 years. The sample comprised two primigravidas, two participants with two pregnancies, three participants with three pregnancies, and three participants with four or more pregnancies. In terms of parity, there were two nulliparous women (no previous births), three participants with one previous birth, three participants with two previous births, one participant with three previous births, and one participant with four previous births. The estimated gestational age (EGA) at booking ranged from 18 to 26 weeks, with a mean of 21 weeks. The distribution showed two participants booked at 18 weeks (P4, P7), three participants at 20 weeks (P1, P6, P10), one participant at 21 weeks (P9), two participants at 22 weeks (P2, P5), one participant at 23 weeks (P3), and one participant at 26 weeks (P8). The educational attainment of participants varied, with three participants having completed Grade 11 (secondary education: P1, P4, P10), five participants having completed Matric (completed secondary education: P2, P3, P5, P6, P8), one participant holding a Diploma (post-secondary: P7), and one participant holding a bachelor’s degree (tertiary education: P9). Participants’ demographic data are summarised in Table 1.

**TABLE 1:** Participants’ demographic data.

Participants code	Age (years)	Gravidity	Parity	EGA at booking	Education level
P1	18	01	00	20	Grade 11
P2	25	02	01	22	Matric
P3	23	01	00	23	Matric
P4	34	04	03	18	Grade 11
P5	37	03	02	22	Matric
P6	30	03	02	20	Matric
P7	33	04	01	18	Diploma
P8	38	05	04	26	Matric
P9	29	02	01	21	Bachelors
P10	31	04	02	20	Grade 11

EGA, estimated gestational age.

Although the participants were somewhat aware of ANC booking and the benefits attached, they brought to light the patient-related, ANC routine, and midwives-related factors as hindrances to seeking early ANC. Three themes with sub-themes emerged. In Theme 1 were patient-related factors which were knowledge deficit, unplanned pregnancies, misconceptions, and experience of ANC. Theme 2 detailed ANC routine factors which comprised fear of mandatory ANC diagnostic tests and increased waiting times. Theme 3 detailed midwives-related factors which included the attitude of midwives and stigmatisation of patients.

A summary of the themes and sub-themes is depicted in Table 2.

**TABLE 2:** A summary of the themes and subthemes.

Theme	Sub-themes
1. Patient-related factors	1.1. Knowledge deficit about pregnancy and antenatal care 1.2. Unplanned pregnancy and denial of the reality of pregnancy 1.3. Misconceptions regarding ANC pharmacological treatment 1.4. Patients’ experiences of ANC
2. Antenatal care routine-related factors	2.1. Fear of mandatory antenatal diagnostic tests (HIV) 2.2. Fear of confirmation of deleterious pregnancy outcomes 2.3. Increased antenatal care waiting time
3. Midwives-related factors	3.1. Attitude of the midwives 3.2. Stigmatisation of patients’ conditions

ANC, antenatal care; HIV, human immunodeficiency virus.

### Theme 1: Patient-related factors

Patient-related factors emerged as significant determinants influencing ANC engagement in our study. These factors encompass a spectrum of personal elements ranging from participants’ baseline knowledge about pregnancy to their individual experiences with healthcare services. This theme revealed how knowledge gaps, pregnancy intentionality, treatment misconceptions and previous experiences collectively shape participants’ decisions and behaviours regarding ANC. The patient-related factors are described under four sub-themes that emerged. The first sub-theme entailed the pregnant women’s knowledge deficit about pregnancy and ANC. The second sub-theme detailed pregnant women’s unplanned pregnancy and denial of the reality of pregnancy. Under the third sub-theme, the pregnant women’s misconceptions regarding ANC pharmacological treatment were detailed. The last sub-theme entailed the patients’ experiences of ANC. The sub-themes explore these dimensions in detail below.

#### Sub-theme 1.1: Knowledge deficit about pregnancy and antenatal care

Under this sub-theme, the knowledge and skills deficit about pregnancy and ANC were detailed. The participants seemed not to have limited knowledge about the importance of ANC and its effect on pregnancy outcomes which influenced their decision to initiate ANC. The participants admitted to their own knowledge deficit regarding pregnancy and childbirth:

‘I don’t think we have enough knowledge about these things [*pregnancy and childbirth*]. If I knew what I know [*now*] about coming to the clinic, I was going to book very early.’ (P3, 23 years, Primigravida, Matric)

The participants were thankful to be attending the ANC and regarded the discussions and health education as eye-opening:

‘I have learnt so much about pregnancy during my visits here.’ (P1, 18 years, Primigravida, Grade 11)

#### Sub-theme 1.2: Unplanned pregnancy and denial of the reality of pregnancy

The participants expressed that the reason they sought ANC services in the advanced stages of their pregnancy was because the pregnancies were unplanned. Therefore, they were unaware of pregnancy and were alarmed by the pregnancy symptoms. The participants explained:

‘I didn’t know that I’m pregnant, so I only found out late that I’m pregnant.’ (P3, 23 years, Primigravida, Matric)

I was still in shock that I am pregnant and really could not concentrate on all other things. (P1, 18 years, Primigravida, Grade 11)

The participants were oblivious to the pregnancy symptoms mainly because they were using contraceptives and were aware of amenorrhoea as a side effect of contraceptives. Participants elaborated:

‘This time I was preventing, so I didn’t know [*that I was pregnant*] because I was preventing, and I was told at the clinics that it is normal to not have my periods. I honestly didn’t know what to expect.’ (P8, 38 years, Gravida 5 para 4, Matric)

‘I last saw my periods in April. Yeah. But then I was like, ah, that’s how the prevention works. Because sometimes I’ll just stay three months to two months without seeing my periods.’ (P5, 37 years, Gravida 3 para 0, Matric)

The participants highlighted that it was not easy to acknowledge their unplanned pregnancies because they were afraid of their elders at home. The participants expressed that seeking ANC services would be confirmatory to the parents, and that was a reality they were ill-prepared to face:

‘For others is because they want to hide the pregnancy as they are afraid that at home, they will see them. It’s the same case with me, with my first pregnancy I was afraid to go to the clinic because of home. I went but I made sure to hide the book so that they do not find it.’ (P2, 25 years; Gravida 2 para 1, Matric)

The participants preferred to even wait for the suggestive signs of pregnancy, to ascertain that they are pregnant before consulting the ANC providers. The participants were unaware of their estimated gestational age and, therefore, they sought the services late in their pregnancies:

‘I had told myself that I want to wait for the obvious signs of pregnancy which is my belly growing. However, I realised that I do not know when I fell pregnant nor how many months [*or*] weeks am.’ (P6, 30 years, Gravida 3 para 2, Matric)

#### Sub-theme 1.3: Misconceptions regarding antenatal care pharmacological treatment

The participants’ knowledge deficit was accentuated by the misconceptions they had about the haematinics and pregnancy supplements. The participants expressed that they avoided the clinic mainly because they had no intention of taking supplements because they believed that they were a source of weight gain. Participants explained:

‘I’m afraid of those pills, they make people sick and all those things.’ (P5, 37 years, Gravida 3 para 0, Matric)

‘I was scared because I did not want to drink their pills. Yeah, I know. People said they make them fat because they make the baby grow too fast and all those things. So, I didn’t want to be too big.’ (P3, 23 years, Primigravida, Matric)

#### Sub-theme 1.4: Patients’ experience of antenatal care services

The primigravida women pointed out that they lacked experience in ANC and were afraid of the unknown which contributed to their delay in seeking ANC. The participants explained:

‘Then I was told to come to the clinic which also needed me to think about it because I didn’t know what was going to happen.’ (P1, 18 years, Primigravida, Grade 11)

On the other hand, the multipara patients narrated their painful previous experiences seeking ANC services. The participants expressed that they were afraid to relive such experiences and avoided the antenatal clinics. The participants explained:

‘With my previous pregnancy, I came here and haven’t felt my baby kicking and the sister [*Midwife*] said she could see the baby’s heartbeat when she was checking me. It was difficult to come back to this clinic.’ (P4, 34 years; Gravida 4 para 3, Grade 11)

Other participants experienced limited access to the ANC services. The participants reported that the BANC services are rendered during the week which is an inconvenience for them because they were employed and needed to be at work during weekdays. Participants elaborated:

‘I was working during the week and over the weekend the clinic does not open.’ (P6, 30 years, Gravida 3 para 2, Matric)

The participants further expressed that absenting themselves from work to attend the ANC services including subsequent visits had a negative impact on their remuneration, as they were not paid for time off work. Participants explained:

‘Even now I find myself thinking that because I am employed now those days when I will be booked for my subsequent visits, they won’t pay me.’ (P5, 30 years, Gravida 3 para 2, Matric)

### Theme 2: Antenatal care routine-related factors

Under this theme, it became evident that the ANC routine-related factors significantly influenced women’s willingness to engage with ANC services. The participants raised emotional and psychological concerns about screening protocols, potential diagnostic outcomes, and the practical realities of service delivery. The participants’ fear was surrounding certain diagnostic procedures and they were anxious about receiving unfavourable pregnancy results. Lastly, the participants were frustrated with facility waiting times. Under this theme, the following three sub-themes emerged. The first sub-theme was about the women’s fear of mandatory antenatal diagnostic tests; the second theme was about the fear of confirmation of deleterious pregnancy outcomes, and the last sub-theme was about the increased ANC waiting time. The sub-themes as interconnected factors are discussed in greater detail below.

#### Sub-theme 2.1: Fear of mandatory antenatal diagnostic tests (HIV)

The participants admitted that they were aware of some of the necessary diagnostic tests performed during ANC. The participants were not ready to learn about their human immunodeficiency virus (HIV) statuses as they fell pregnant before undergoing voluntary counselling and testing for HIV. Participants elaborated:

‘I was scared that they were going test me for HIV, I was really scared to find out about my HIV status because it was my first pregnancy.’ (P3, 23 years, Primigravida, Matric)

Furthermore, procedures such as the collection of blood for pregnancy-related investigations were stressful and fear-generating for the participants. Participants mentioned:

‘I’m afraid … they’ll take your blood every three months. So, that’s the problem.’ (P5, 37 years, Gravida 3 para 2, Matric)

#### Sub-theme 2.2: Fear of confirmation of deleterious pregnancy outcomes

Participants revealed that they were terrified of finding out about the conditions of foetuses and their general conditions. The participants were weary of intrauterine foetal deaths (IUFD), miscarriages or something wrong with the pregnancy. The participants revealed:

‘Personally, I got many miscarriages that I do not know were caused by what, so you don’t even know what the nurses are going to say about the pregnancy when you get to the clinic.’ (P7, 33 years, Gravida 4 para 1, Diploma)

‘When I came here, the sister said I have to go to the hospital for the sonar because I didn’t know my dates [*last normal mensuration*], when I got there the doctor said there was something wrong with [*the*] baby inside [*Gross congenital abnormalities*] and it is best that I don’t continue with the pregnancy.’ (P10, 31 years, Gravida 4 para 2, Grade 11)

#### Sub-theme 2.3: Increased antenatal care waiting time

The participants highlighted the prolonged waiting time in the clinical facilities as another reason for avoiding the clinical facility. The participants explained that they needed to be at the ANC clinics from the early hours of the morning and had to dedicate most of the day to being at the antenatal facility. The participants elaborated:

‘This thing of waking us early, 05:00, while the clinic is still closed. No, it must be cancelled. No, we can’t.’ (P5, 37 years, Gravida 3 para 2, Matric)

‘Around 09:00 [*or*] 10:00, you are still here.’ (P6, 30 years, Gravida 3 para 2, Matric)

Furthermore, participants highlighted that they needed to be early to access the services to avoid being returned. Participants elaborated:

‘That’s how it works. If you don’t book the line, then you are the last one. Also, you … if you come around after 08:00, you are the last one. And you are the latecomer then other clinics, they will not even attend or do anything to you if you come late.’ (P5, 37 years, Gravida 3 para 2, Matric)

### Theme 3: Midwives-related factors

Midwives-related factors emerged as a significant theme affecting participants’ engagement with antenatal services. The professional demeanour of midwives and their responses to participants’ various conditions created lasting impressions that hindered future care-seeking behaviours. Two sub-themes emerged in this theme. The first sub-theme was the attitude of midwives towards the pregnant women. The second sub-theme was the stigmatisation of patients’ conditions. The two sub-themes explore how midwives’ attitudes and tendencies towards stigmatisation impacted women’s willingness to access antenatal services.

#### Sub-theme 3.1: Attitude of the midwives

The participants explained that the attitude of midwives is the reason for their choices to avoid the ANC clinics. The participants expressed that they had witnessed the negative attitude projected by the midwives and preferred to attend private practitioners. The participants detailed:

‘Only one reason, the nurses’ attitude. Personally, I was not, uhm, like I said if the nurses were rude, I was not going to go anymore. I was going to stay at home, probably continue going to the doctor or find another way to get through at this.’ (P9, 29 years, Gravida 2 para 1, Bachelors)

‘And they’ll always shout, they won’t become understanding. So, most of the women are afraid of that.’ (P10, 31 years, Gravida 4 para 2, Matric)

#### Sub-theme 3.2: Stigmatisation of patients’ conditions

The participants were troubled by the stigmatisation they experienced from the midwives. The participants were of the opinion that midwives treated patients with HIV differently. The HIV-positive pregnant women feared stigmatisation and refrained from seeking ANC services. The participants elaborated:

‘The sisters neh, they’ll treat you like you are HIV-positive. Maybe you are dying. So, that’s the reason.’ (P8, 38 years, Gravida 5 para 4, Diploma)

The participants were of the opinion that the midwives were over-controlling; consequently, the participants felt that they were excluded from their own plans of care. The participants explained:

‘They’ll be like, “Why don’t you do this? Why don’t you do that?”’ (P2, 25 years, Gravida 2 para 1, Matric)

‘They were shouting, telling you how to live your life and all those things, but I managed to call them down. So, most of the women are afraid. They [*patients*] don’t want to be controlled.’ (P8, 38 years, Gravida 5 para 4, Matric)

## Discussion

The study revealed several critical insights into the factors influencing late antenatal booking among pregnant women in the Tshwane district. The key findings include significant barriers that prevent early and successful booking for ANC. Globally, the ANC is regarded as a preventative tool to detect early and manage pregnancy-related conditions that may cause deleterious patient outcomes.^3^

Significant barriers exist that prevent early and successful booking for ANC. There is a notably slow uptake of antenatal services, despite global recommendations. Multiple complex factors contribute to delayed ANC engagement.

The findings of this study revealed a knowledge deficit regarding reproductive health. The participants reported that they fell pregnant while using contraceptives. This finding may suggest a lack of in-depth understanding of the effective use of contraceptives, which substantiates studies that found that, although people within childbearing age were aware of the variety of contraceptive methods, most people lacked an understanding of the mechanisms of actions of such contraceptives.^28^ Unfortunately, this finding may also suggest that there is less adherence to the recommended dual method of protection against pregnancy, sexually transmitted infections (STIs), and HIV, consequently, placing patients at an even higher risk of contracting HIV and STI.^29^ In addition, this finding could be associated with the rate of unplanned pregnancies reported by participants as a factor that hindered early initiation of ANC.

The misconceptions regarding the necessary supplementation of pregnancies also evidenced the participants’ knowledge deficit. Most participants believed that macronutrient supplements were responsible for exaggerated weight gain during pregnancy. This finding substantiates the already existing non-adherence to treatment among the women, which unfortunately suggests that the participants are unaware of the risk of potential anaemia and of giving birth to neonates with neural tube defects.^30^

The lack of previous experience of ANC was associated with the reluctance to initiate ANC. The primigravida with less experience in ANC were terrified to initiate the care and feared the activities of the care. In addition, the participants expressed that they needed to be made aware of what to expect. This finding substantiates the well-documented fear of unknown pregnancy and childbirth events among pregnant women.^31^ Fear of pregnancy and childbirth is among the first times the myths and terrifying tales exacerbate pregnant women’s experiences mothers share. Consequently, the first-time pregnant women delay seeking ANC services.^31^

The procedural diagnostic tests performed during ANC, such as HIV counselling and testing, emerged as a key finding and one of the reasons they were wary of seeking ANC services. Unfortunately, the finding suggests that the participants’ pregnancies were unplanned, considering that HIV counselling forms part of preconception care.^32^ This finding confirms the findings of studies on the prevention of mother-to-child transmission, which found that women test and confirm their HIV status for the first time during pregnancy.^33^ Consequently, to avoid learning about HIV status, initiation of ANC was delayed.^34^

A key finding was that previously experienced pregnancy-related complications were a source of fear, as the diagnostic procedures might confirm their fear of the presence of a certain complication. This finding is valid because some of the previous complications of pregnancy such as pre-eclampsia and placenta abruptio may recur in subsequent pregnancies.^35^ Unfortunately, this finding may also suggest that patients with previous pregnancy-related complications may be suffering from unresolved post-traumatic stress disorder (PTSD) which requires attention to avoid complications. According to the existing literature, unresolved PTSD may complicate antenatal depression which already affects about 7% – 20% of pregnancies globally.^36,37^ Unfortunately, with pregnant women reporting late for ANC, the PTSD and antenatal depression may go unnoticed and present as postpartum depression (PPD).^36^

The previously experienced prolonged waiting times in the ANC facilities emerged as one of the factors that delayed seeking this essential service. The increased waiting time in the clinical facilities emerged as a contributing factor to pregnant women avoiding seeking ANC services. The increased waiting time is a long-standing problem within the public clinical settings.^37^ The literature highlights that patients spend more time than is necessary waiting for clinical assistance in healthcare facilities.^38,39^ Consequently, the DOH has prescribed a waiting time of at least 2 h for patients in primary health care facilities.^38^ Unfortunately, the increase in waiting time emerges as a factor that demoralises the patients from seeking ANC services. This finding suggests that late ANC bookings and non-booked clients remain a challenge in reaching 100% targets in ANC compliance according to BANC guidelines.^39^

The negative attitude of midwives emerged as a hindrance to ANC services, which made pregnant women wary of seeking ANC services. The attitude of healthcare providers is a challenge within public healthcare facilities and has resulted in the South African Nursing Council (SANC) documenting a code of ethics for nursing practice, and the DOH laying down patient safety as one of the key priorities.^40^ This finding suggests that while midwives aim to improve the health outcomes of pregnant women and their unborn babies, they need to be cautious not to alienate pregnant women from the ANC service.^41^ Some of the participants reported they have previously experienced stigmatisation based on their clinical conditions and history such as positive HIV status, gravidity and parity. Stigma is reported as a hindrance to accessing healthcare services; a similar effect of stigma is reported even in the Lesbian, Gay, Bisexual, Transgender, Queer/Questioning, Intersex, and Asexual/Ally (LGBTQIA) context.^42^ The primary health care nurses were found to be judgemental and demonstrated less interest in meeting the specific health needs of the patient who identified as LGBTQIA.^42^ The identified factors that hinder pregnant women from seeking ANC services as soon as the pregnancy is diagnosed could be the reason for patients’ inappropriate responses to pregnancy.

### Strengths and limitations

The study employed a qualitative research approach that centred on the perceptions of pregnant women who booked late for ANC, offering a unique and valuable perspective on service utilisation. By prioritising the voices of primary ANC service consumers, the research enabled participants to articulate their personal experiences and reasons for delayed booking, thereby providing rich, contextual insights into the factors influencing their healthcare-seeking behaviours. This participant-centred methodology allowed for an in-depth exploration of individual perceptions that quantitative methods might overlook, simultaneously offering an opportunity for quality improvement by highlighting systemic barriers from the perspective of those directly affected.

However, the study acknowledges several methodological limitations that warrant careful consideration. The research was constrained by a small sample size of 10 participants, focused exclusively on three facilities in the Tshwane district, which significantly limits the generalisability of the findings. By concentrating solely on pregnant women’s perspectives, the study potentially missed complementary insights that could have been provided by healthcare providers such as midwives. The highly localised nature of the research necessitates cautious interpretation when considering broader applications.

Readers are advised to approach the findings as rich, exploratory insights rather than definitive conclusions. The study’s value lies in its ability to provide a nuanced understanding of late ANC booking factors, serving as a foundational piece of research that invites further investigation. Future research could address the current limitations by expanding the sample size, incorporating multiple perspectives, and conducting comparative studies across different geographical and cultural contexts. Despite these limitations, the study’s participant-centred qualitative methodology offers a critical lens through which to understand the complex dynamics of ANC service utilisation.

## Conclusion

The study reveals critical insights into the barriers preventing early ANC booking, identifying several significant gaps in current healthcare service delivery. The key identified barriers include limited health education, misconceptions about pregnancy care, and potential communication challenges between healthcare providers and pregnant women.

The findings underscore the urgent need for comprehensive interventions targeting multiple aspects of ANC. Specifically, midwives are called upon to enhance health education strategies, with a particular focus on reproductive health and preconception care. This approach aims to reduce unintended pregnancies and improve overall maternal healthcare engagement. Moreover, the research highlights the importance of demystifying pregnancy-related myths surrounding diagnostic tests, medical interventions, and pharmacological management.

A crucial recommendation emerging from the study is the necessity for midwives to engage in critical self-reflection. This involves creating a therapeutic, non-threatening healthcare environment that encourages women to seek and continue antenatal services. By addressing communication barriers and improving patient-provider interactions, healthcare providers can potentially increase early ANC booking rates.

The study’s goal aligns with global maternal health objectives: to ensure that pregnant women initiate antenatal services before 12 weeks of gestation and attend at least eight ANC visits throughout their pregnancy. The identified factors serve as a valuable guide for policymakers to restructure and improve preconception and ANC services, ultimately aiming to maintain healthy pregnancies and minimise adverse patient outcomes. These recommendations represent a strategic approach to addressing systemic challenges in maternal healthcare, emphasising the need for patient-centred, compassionate and comprehensive care.

### Recommendations and implications

The study’s findings reveal critical implications for nursing practice, education, research and policy, necessitating a comprehensive and collaborative approach to addressing late ANC booking. In nursing practice, midwives are called upon to redefine their role as primary community health educators, developing strategies that create supportive, non-threatening healthcare environments. This involves enhancing communication approaches, building patient trust and implementing targeted interventions that address patients’ potentially inappropriate responses to pregnancy.

From an educational perspective, there is an urgent need to redesign reproductive health curricula for healthcare professionals. Educational programmes must focus on comprehensive preconception care, integrating targeted strategies for different age groups, particularly teenagers. The emphasis should be on developing healthcare communication training that incorporates myth-busting techniques and promotes culturally sensitive, patient-centred health education. This approach requires creating innovative training modules that enhance skills in early pregnancy detection and holistic care.

Research implications demand a multi-faceted investigation into the complex factors preventing early antenatal booking. Researchers are encouraged to develop and test intervention strategies, explore community-specific barriers, and create comprehensive research frameworks that prioritise patient perspectives. Comparative studies across diverse demographic and geographical contexts will be crucial in understanding the nuanced challenges surrounding ANC service utilisation.

Policy recommendations call for a systematic review of current ANC access policies. Policymakers must develop comprehensive strategies that improve ANC service accessibility, establish guidelines for community-based reproductive health awareness, and allocate resources for targeted health education programmes. The goal is to create policy mechanisms that support early pregnancy detection and care, ultimately reducing unintended pregnancies and improving maternal health outcomes.

The overarching strategic approach emphasises a holistic, integrated methodology that transcends traditional healthcare boundaries. By synchronising efforts across nursing practice, education, research and policy domains, healthcare systems can develop proactive, patient-centred strategies. These interventions aim to empower individuals with knowledge, reduce healthcare barriers and create supportive environments that facilitate comprehensive maternal care. The ultimate objective remains improving maternal and child health outcomes by addressing the root causes of late ANC booking.
